# Neural Codes for One’s Own Position and Direction in a Real-World “Vista” Environment

**DOI:** 10.3389/fnhum.2018.00167

**Published:** 2018-04-30

**Authors:** Valentina Sulpizio, Maddalena Boccia, Cecilia Guariglia, Gaspare Galati

**Affiliations:** ^1^Department of Psychology, “Sapienza” University of Rome, Rome, Italy; ^2^Cognitive and Motor Rehabilitation Unit, Fondazione Santa Lucia (IRCCS), Rome, Italy

**Keywords:** spatial representation, individual differences, hippocampus, retrosplenial complex, parahippocampal place area, occipital place area, fMRI adaptation, multi-voxel pattern analysis

## Abstract

Humans, like animals, rely on an accurate knowledge of one’s spatial position and facing direction to keep orientated in the surrounding space. Although previous neuroimaging studies demonstrated that scene-selective regions (the parahippocampal place area or PPA, the occipital place area or OPA and the retrosplenial complex or RSC), and the hippocampus (HC) are implicated in coding position and facing direction within small-(room-sized) and large-scale navigational environments, little is known about how these regions represent these spatial quantities in a large open-field environment. Here, we used functional magnetic resonance imaging (fMRI) in humans to explore the neural codes of these navigationally-relevant information while participants viewed images which varied for position and facing direction within a familiar, real-world circular square. We observed neural adaptation for repeated directions in the HC, even if no navigational task was required. Further, we found that the amount of knowledge of the environment interacts with the PPA selectivity in encoding positions: individuals who needed more time to memorize positions in the square during a preliminary training task showed less neural attenuation in this scene-selective region. We also observed adaptation effects, which reflect the real distances between consecutive positions, in scene-selective regions but not in the HC. When examining the multi-voxel patterns of activity we observed that scene-responsive regions and the HC encoded both spatial information and that the RSC classification accuracy for positions was higher in individuals scoring higher to a self-reported questionnaire of spatial abilities. Our findings provide new insight into how the human brain represents a real, large-scale “vista” space, demonstrating the presence of neural codes for position and direction in both scene-selective and hippocampal regions, and revealing the existence, in the former regions, of a map-like spatial representation reflecting real-world distance between consecutive positions.

## Introduction

In everyday life, our ability to keep oriented in the world depends on the accurate estimation of two spatial features: one’s own location and facing direction. Neurophysiological evidence on freely moving animals reveals the existence of specific cells that encode these spatial information: place cells in the hippocampus (HC), which fire as a function of the spatial position independently of the animal’s facing direction (O’Keefe and Dostrovsky, 1971), and head-direction cells in Papez circuit structures, which fire on the basis of the facing direction independently of the animal’s location (Chen et al., 1994; Taube, 1998).

Recent neuroimaging evidence revealed that a similar navigational system is implemented in humans. By combing different analyses approaches (adaptation and multi-voxel pattern analyses) on functional magnetic resonance imaging (fMRI) data, we recently demonstrated that scene-selective regions such as the parahippocampal place area (PPA) and the retrosplenial complex (RSC) automatically encode one’s own position and direction within a familiar virtual room and that these spatial quantities are organized as a map: similar activity patterns were observed for locations closer in physical space (Sulpizio et al., 2014). A quite different organization was instead observed in a previous study exploring the neural coding of position and direction within a large-scale, real-world environment (Vass and Epstein, 2013). The authors, by taking advantage of multi-voxel pattern analysis to explore the selectivity towards location and heading direction within a large-scale, real-world environment, found that the pattern of activity in RSC contains information about location but not about facing direction. Additionally, the authors failed in finding a relationship between real-world distances between locations and the activity patterns of PPA, RSC and any other brain regions. However, by using the same environment (the Pennsylvania University campus), Morgan et al. (2011) revealed a map-like spatial organization in the human HC. When participants viewed images of familiar campus buildings, the hippocampal activity response to each building scaled with the distance between that building and the building shown on the immediately preceding trial. Similar results were also obtained when requiring individuals to navigate through a complex real-world space such as a city district with many interconnected streets. For example, Howard et al. (2014) reported that the posterior hippocampal activity was sensitive to the path distance to the goal during navigation within the London’s Soho district and that this distance-related effect was abolished when travel was guided by external cues. Map-like codes in the human HC have also been identified after learning spatio-temporal trajectories in a large-scale space. Specifically, Deuker et al. (2016) demonstrated that neural similarities in the hippocampal patterns reflected the remembered proximity of events within large-scale virtual city in both time and space.

Insight into the existence of a distance-dependent representation comes from behavioral and imaging studies exploring spatial memory through table-top or virtual room-sized displays. Performance decreased linearly with the amount of viewpoint rotation when asking participants to recall the object-to-object spatial relationship on a table (Diwadkar and McNamara, 1997), and to retrieve target locations across different perspectives in a familiar virtual room (Sulpizio et al., 2013, 2015, 2016b). Imaging evidence also reported orientation-dependent effects in scene-selective regions. For example, PPA and RSC showed an increase of activation as a function of the amount of experienced view change (Schmidt et al., 2007; Sulpizio et al., 2013), although only the RSC activity scaled with the size of viewpoint changes in the environmental frame (Sulpizio et al., 2013).

To summarize, previous works suggested that the human brain automatically encodes the recovered position and facing direction within the environment, although the existence of a map-like representation of these spatial codes is not consistent across these studies. It is possible that differences in the experimental settings may account for such a discrepancy. For example, some studies have used small-scale room-sized virtual environments (Schmidt et al., 2007; Sulpizio et al., 2013, 2014, 2015, 2016b) or table-top displays in real environments (Diwadkar and McNamara, 1997) in order to tightly control exposure to specific directions. In such situations, the observer could apprehend discrete locations from a single standpoint without remarkable locomotion (“vista” space). Other studies have used large-scale real environments (Morgan et al., 2011; Vass and Epstein, 2013; Howard et al., 2014) or large virtual spaces (Marchette et al., 2014), in which target locations, being beyond the sensory horizon, could only be represented after integrating multiple views acquired during locomotion (“environmental” space). Thus, disentangling between “vista” and “environmental” spaces seems to be essential, especially in the context of navigation (Montello, 1993; Wolbers and Wiener, 2014). However, the impact of different spatial scales on spatial representations of one’s own position and direction has been rarely considered. The available imaging evidence speaks in favor of a significant impact of the spatial scale on these spatial quantities, with a metric, map-like spatial organization mainly observed in the HC during passive viewing of familiar buildings or during navigation within large-scale environments (Howard et al., 2014; Deuker et al., 2016), and in PPA and RSC during the mere exposure to small-scale room-sized, virtual environment (Sulpizio et al., 2014) or during object memory tasks within it (Schmidt et al., 2007; 2013, 2016b).

However, although the “vista” vs. “environmental” distinction is crucial in the context of navigation, “vista” and “environmental” spaces are not necessarily different in terms of scale of space. For example, a single room or a space with multiple corridors may be equivalent in terms of spatial scale but different in terms of target visibility. Thus, since the small- vs. large-scale distinction does not necessarily coincide with the “vista” vs. “environmental” dichotomy, in the present study we sought to clarify the impact of the scale of space on spatial representations after controlling for this aspect. To do this, we focused on the neural representation elicited by the “vista” space so as to indirectly test the effect of spatial scale on position- and direction-dependent representations by exploring whether the neural code for one’s own position and direction within a large open-field (“vista”) space, such as a real town square, reveals the same organization previously observed in a smaller room-sized “vista” environment (Sulpizio et al., 2014). Although we recently reported behavioral priming for repeated positions and directions within that environment, with priming effects scaling with the real-word distances between these spatial quantities (Sulpizio et al., 2017), it remains unclear how such a space is represented in the human brain.

We asked participants to observe consecutive images, which varied for position and facing direction within a familiar real-world circular square. We hypothesized that both position and facing direction within such a “vista” space is represented in the HC and in scene-selective regions. To test this hypothesis, we combine functional magnetic imaging adaptation effects (Grill-Spector et al., 2006) elicited by repetition of any of the two spatial quantities across consecutive pictures, and multivariate pattern analysis (MVPA) to determine which information elicit patterns that are distinguishable (Morgan et al., 2011; Epstein and Morgan, 2012). Following Drucker and Aguirre (2009) hypothesis, these two techniques should explore different aspects, with the former reflecting clustering at a coarser spatial scale while the latter revealing tuning of individual neurons. We hypothesize that all the above-mentioned regions should be clustered according to the (implicitly) encoded spatial information, thus permitting decoding of both positions and directions using multi-voxel patterns. This approach identifies neural patterns which are consistently associated with one position/direction over time, i.e., neural “signatures” of long-term memory traces. On the other side, since univariate analysis of adaptation effects would be instead sensitive to the relationship between consecutive trials, thus reflecting the effect of “being in the same place as before” vs. “being in a different place”, but irrespective of the absolute spatial location, we expected to find a more specific involvement of the explored regions in the dynamic process of updating these spatial information.

Beyond PPA and RSC, we also explored the role of the occipital place area (OPA). Although little is known about its function, recent work suggests that OPA supports navigation guided by visual cues and representation of local elements in the immediately visible scene, such as obstacles (Kamps et al., 2016), as well as the encoding of navigationally-relevant information such as environmental boundaries (Julian et al., 2016) and local navigational affordances (Bonner and Epstein, 2017). Additionally, inspired by previous evidence of a map-like representations for positions and facing directions (Sulpizio et al., 2014, 2017), we further explored whether hippocampal and scene-selective regions support this metric code even in a real-world “vista” space. We tested this hypothesis, by analyzing neural adaptation effects as a function of real-world distances between the covered positions/directions in consecutive images.

Another important aspect to be considered in the context of navigation is the individual experiential level: in some of the above-described studies participants learned a new environment (Schmidt et al., 2007; Sulpizio et al., 2013, 2014, 2015, 2016b; Marchette et al., 2014), typically through a limited number of exposures, while in other studies (Morgan et al., 2011; Vass and Epstein, 2013; Howard et al., 2014) they were very familiar with the experimental layout which was learned over extended time periods (typically years). One possibility is that the degree of familiarity with the environment affects the organization of position- and direction-dependent representations, and thus account for the reported discrepancy in the literature. We tested this hypothesis by controlling for the individual *a priori* and global knowledge of the environment, by using a series of questionnaires and training tests.

Finally, corollary to these aims, we further explored the impact of individual differences, in terms of navigational ability, on the neural representation of position and direction in both hippocampal and in scene-selective regions. Previous imaging studies have demonstrated that poor navigators showed a lower accuracy at identifying the most stable landmarks in the scene, and exhibited reduced responses in RSC, as compared to good navigators (Auger et al., 2012). To test this hypothesis we administered a self-reported questionnaire of navigational ability, the Santa Barbara Sense of Direction (SBSOD) questionnaire (Hegarty et al., 2002) that has been shown to be a reliable instrument to predict performance on objective tasks requiring to update one’s location and direction in the environment (Kozhevnikov and Hegarty, 2001). Specifically, we expected that the individual differences in navigational abilities interact with the RSC function of encoding spatial information.

## Materials and Methods

### Participants

Eighteen neurologically normal volunteers (9 females, mean age 27 s.d. 2.54) participated in the study. Sample size was determined based on previous fMRI experiments on the same topic (for a meta-analysis, see Boccia et al., 2014). One participant was excluded because he took part only to one of the two fMRI sessions. All participants were right handed, as assessed by the Edinburgh Handedness Inventory (Oldfield, 1971) and had normal or corrected-to-normal vision. All volunteers gave their written informed consent to participate in this study, which was approved by the local research ethics committee of the IRCCS Fondazione Santa Lucia in Rome, according to the Declaration of Helsinki.

### Stimuli and Task

**Figure 1:**
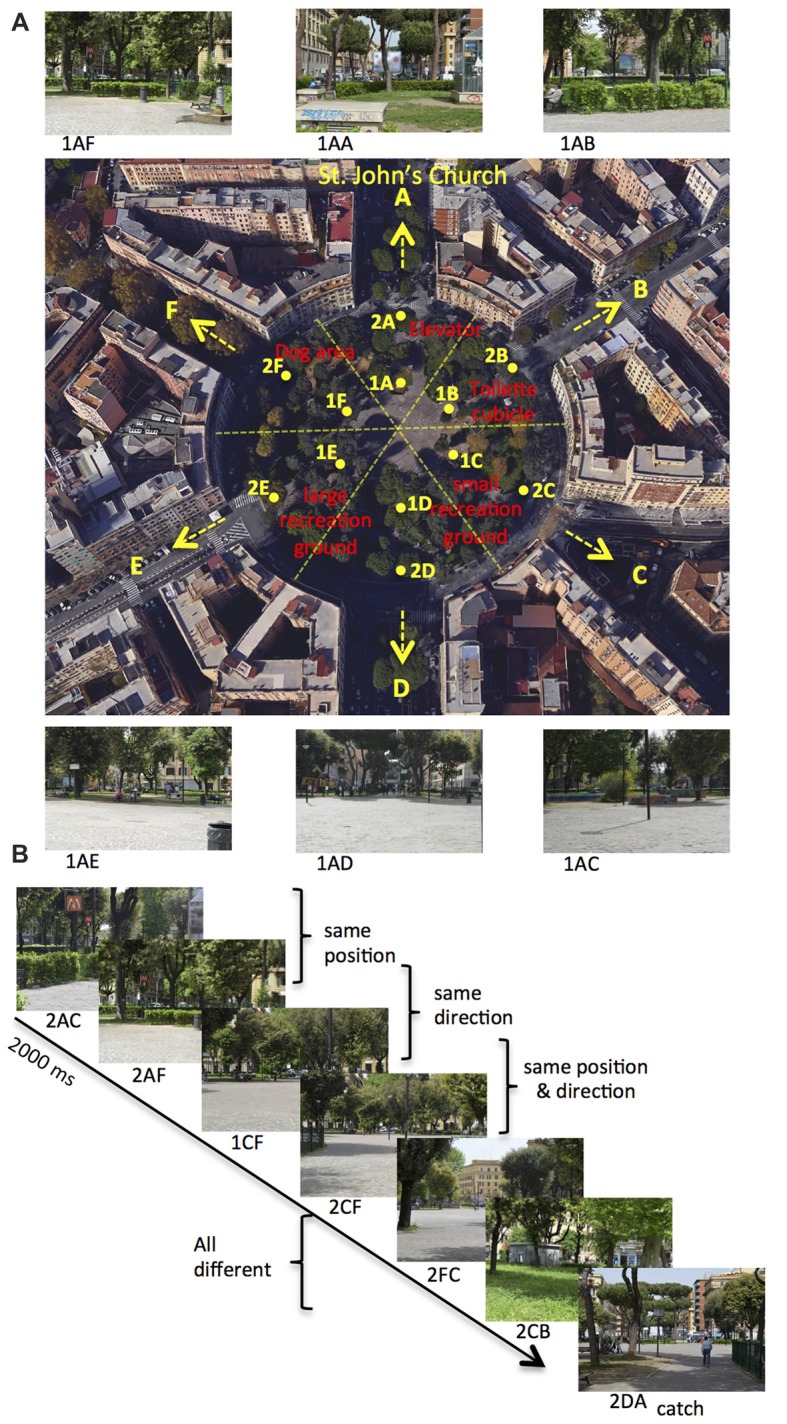
Environment and paradigm. **(A)** Map of Rome Kings’ Square. The six roads departing from the square define the six possible directions (marked as A–F), with the A direction (north) pointing towards St. John’s Church. The square can be ideally divided in six wedges, and the 12 points (A–F, distributed around two concentric circles: eccentricity 1–2) represent all the possible positions within the square. Red labels indicate the relevant landmarks within the square. The label below the photograph (not shown to the participant) identifies the position from which the photograph is taken (first two letters: 1A to 2F) and its facing direction (third letter: A–F). **(B)** Example of trial sequence. Participants were presented a series of pictures and they were instructed to press a button, except when the presented picture was taken from the A direction (catch trial). Trial stimuli show the same position, the same direction or the same position and direction as compared to the previous trial.

We used the same stimuli used in Sulpizio et al. (2017). We acquired each stimulus, consisting of a digitized color photograph (1024 × 768 pixel resolution), from one out of six different locations within Rome Kings’ Square (*Piazza dei Re di Roma*) in Rome and orientated toward one of the different equidistributed directions starting from the square. Rome Kings’ Square is a large (130 m of diameter), radial-arm maze-like round square situated in the Appio Latino neighborhood, distant 750 m from the Archbasilica of St. John in the Lateran (Figure 1A). Each photograph describes a specific position and facing direction within the square. Each location (A–F, Figure 1A) corresponds to one of the six wedges in which the square can be ideally subdivided. Within each wedge, we acquired photographs from two different positions (1–2) located at the distance of 32 m and 64 m from the center of the square, respectively. Each facing direction (A–F, Figure 1A) corresponds to one of the six streets (*Appia—*St. John direction, *Aosta*, *Pinerolo*, *Appia—Pontelungo* direction, *Albalonga* and *Cerveteri*) originating from the square. We thus acquired a total of 72 images (6 locations × 2 distances × 6 directions). Different landmarks are present within the square: a small recreation ground, a large recreation ground, the elevator of the tube station, a toilette cubicle and a dog area. Examples of stimuli taken from a specific position within the square, and with a specific facing direction, are shown in Figure 1A.

In the fMRI acquisition session, participants observed sequences of pictures, each describing a specific position and direction within Rome Kings’ Square. These pictures were presented in a serially-balance sequence (carry-over sequence, see Aguirre, 2007), in which each picture was preceded by every other picture equally often so as to counterbalance main effects and first-order carry-over effects. This was crucial to allow us to use the same stimuli for both univariate (fMR adaptation) and multivariate (MVPA) analyses (Morgan et al., 2011; Epstein and Morgan, 2012). Participants were presented 72 pictures, which varied for the participants’ position (A–F, Figure 1A) and direction (A–F, Figure 1A) within the square. Figure 1B shows an example of a brief sequence of experimental trials. On each stimulus, participants always pressed a button, except when the observed picture was directed toward the St. John’s church (i.e., pictures in the A direction: catch trials). Catch trials were excluded from all the following analyses. We introduced this task to prompt participants to pay attention to all pictures and thus it was incidental to the aim of the study, that is exploring the neural representations of one’s own position and direction within a familiar, real environment.

### Apparatus and Procedure

We acquired images using a 3T Siemens Allegra MR system (Siemens Medical systems, Erlangen, Germany) equipped for echo-planar imaging with a standard head coil and operating at the Neuroimaging Laboratory, Foundation Santa Lucia. Visual stimuli were presented by a control computer located outside the MR room, running in-house software (Galati et al., 2008) implemented in MATLAB (The MathWorks Inc., Natick, MA, USA). We used an LCD video projector with a customized lens to project visual stimuli to a projection screen positioned at the back of the MR tube. Visual stimuli were thus visible by participants through a mirror positioned inside the head coil. The timing of presentation of each stimulus was controlled and triggered by the acquisition of fMRI images. We recorded participants’ responses through push buttons connected to the control computer via optic fibers.

We used blood-oxygenation level-dependent imaging (Kwong et al., 1992) to acquire echo-planar functional MR images (TR = 2 s, TE = 30 ms, flip angle = 70°, 64 × 64 image matrix, 3 × 3 mm in-plane resolution, 30 slices, 4.5 mm slice thickness with no gap, interleaved excitation order) in the AC–PC plane. Images were acquired for all the cerebral cortex, except for the most ventral portion of the cerebellum. For each participant we also acquired a three-dimensional high-resolution anatomical image (Siemens MPRAGE sequence, TR = 2 s, TE = 4.38 ms, flip angle = 8°, 512 × 512 image matrix, 0.5 × 0.5 mm in-plane resolution, 176 contiguous 1 mm thick sagittal slices). For each scan, we discarded the first four volumes in order to achieve steady-state, and the experimental task was initiated at the beginning of the fifth volume.

**Figure 2:**
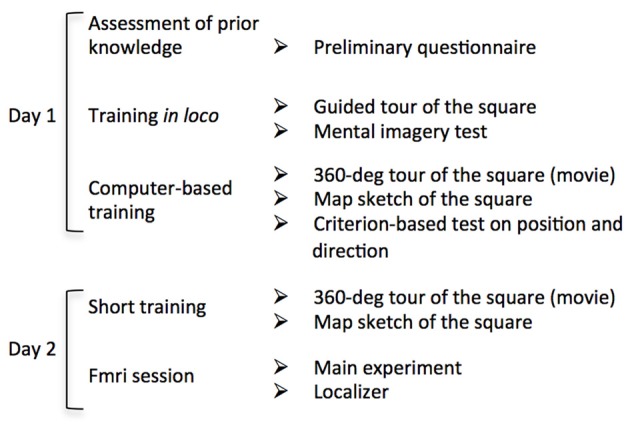
Flow chart of the experimental procedure. Schematic description of each stage of the experimental protocol. On Day 1 participants underwent an intensive familiarization session including both *in loco* and computer-based tasks. On day 2, after a brief familiarization, participants underwent the fMRI session consisting in the main experiment and two “localizer” imaging runs.

The experimental procedure was schematically described in Figure 2. On day one, participants underwent the same familiarization protocol used in Sulpizio et al. (2017). We first administered a preliminary questionnaire to estimate the *a priori* knowledge of the Rome Kings’ Square; we asked participants to report the frequency by which they visit the square (never; one time a year or less; many times a years; many times a month; many times a week; every day). After this preliminary assessment, participants underwent an intensive training session within Rome Kings’ Square aiming at ensuring the development of a long-term knowledge of the square layout. Further, we used a paper-pencil test adapted from Palermo et al. (2012) to assess the ability to build a stable cognitive map of this real environment. Specifically, participants were guided by the examiner through a 360° tour of the square. We asked participants to memorize the landmarks location as well as the six facing directions. Subsequently, they had to describe the environment from their mental imagery by responding to a 5-item questionnaire. For a detailed description of the questionnaire see Sulpizio et al. (2017).

After familiarization in the square, we asked participants to complete a series of computer-based experiments in the laboratory. Before testing, we allowed participants to familiarize again with the environment. We presented a first-person-view movie reproducing a 360° tour of the square. During this period, participants reinforced their memories about the relative locations of the streets (directions) and about landmarks location within the square. We presented the movie until participants were sure to correctly reproduce a sketch depicting the aerial view of the square. All participants reproduced the correct map after observing either one or two virtual tours of the square. We then prompted the individual ability to encode one’s own position and facing direction within the explored environment (Sulpizio et al., 2017) through a training task including a series of questions about the covered position and direction within the square (for the same procedure, see Sulpizio et al., 2017). In each trial, a picture of the square taken from an unpredictable viewpoint was presented (Supplementary Figure S1). This picture included also the schematic sketch of the square (from a survey viewpoint) in which the six wedges and the six arms represented the six possible positions and directions, respectively (Supplementary Figures S1A,B). In separate runs, participants decided whether the covered position in the square matched with the wedge highlighted in the sketch (“position” questions; Supplementary Figure S1A) or whether the perceived direction corresponded to the arm highlighted in the sketch (“direction” questions Supplementary Figure S1B). In case of matching between the experienced position/direction and the highlighted wedge/arm of the sketch, participants were instructed to press the left button on a 2-button response device with their right index; in case of mismatch they were instructed to press the right button with their middle finger. We presented a total of 144 pictures (72 for “position” questions and 72 for “direction” questions). Each picture remained on the screen until participants answered and the next trial started after a fixed inter-trial interval (ITI) of 500 ms. We took advantage of this training phase to force participants to develop a long-term knowledge of the explored environment so that they should be able to encode the current location and direction within the square. In these training sessions each participant had to reach a criterion of at least 70% of accuracy.

On the following day, we scanned participants during an fMRI acquisition session, including the main experiment and a “localizer” experiment. Before starting the experiments, we allowed participants to familiarize with the environment again. A movie reproducing a 360° tour of the square was presented again; we asked participants to watch it as long as they needed to correctly draw the schematic (aerial) view of the square. All participants reproduced the correct map of the explored environment at the first attempt.

The main experiment consisted of six fMRI scans lasting approximately 8 min each (264 functional MR volumes for the first scan and 221 for the remaining five scans), comprising 930 target trials and 31 catch trials, plus 74 randomly intermixed fixation periods each lasting 8000 ms long, providing a baseline. Each trial was presented for 2000 ms, followed by an ITI of 500 ms.

Participants also completed two localizer imaging scans consisting of eight alternating blocks (16 s) of photographs of faces and places/scenes presented for 300 ms every 500 ms, interleaved with fixation periods of 15 s on average (see Sulpizio et al., 2013). During each scan, lasting approximately 7 min (234 functional MR volumes), participants were instructed to passively view each picture. Data from these scans were used to identify scene-responsive regions in the parahippocampal, retrosplenial and occipital cortex (Epstein, 2008).

### Image Processing and Analysis

Images preprocessing and analyses were carried out using SPM12 (Wellcome Department of Cognitive Neurology, London, UK). Functional images were corrected for differences in slice timing by using the middle slice acquired in time as reference; images were spatially corrected for head movements (realignment) by using a least-squares approach and six parameter rigid body spatial transformations. We then coregistered images of each participant onto their anatomical image and spatially normalized using an automatic non-linear stereotaxic normalization routine (final voxel size: 3 mm × 3 mm × 3 mm). For spatial normalization we used a template image based on average data provided by the Montreal Neurological Institute (Mazziotta et al., 1995). Images for univariate analyses were spatially smoothed using a three dimensional Gaussian filter (6 mm full-width-half-maximum for the main experiment and 4 mm full-width-half-maximum for the localizer scans); multi-voxel patterns analyses (MVPAs) were conducted on unsmoothed images.

For each participant we analyzed time series of functional MR images separately on a voxel-by-voxel basis, according to the general linear model (GLM) as implemented in SPM12. We used a temporal high-pass filter in order to remove low-frequency confounds with a period above 128 s and estimated serial correlations with a restricted maximum likelihood (ReML) algorithm; the ReML estimates were then used to whiten the data.

Analyses were conducted on four independently defined, theoretically motivated, regions of interest (ROIs). Three of them, i.e., the PPA, the RSC and the OPA were identified on each individual’s cortical surface (segmented by using an automatic procedure as implemented in Free-Surfer software package) by analyzing data from the “localizer” scans in which place/scene and face blocks were modeled as box-car functions, convolved with a canonical hemodynamic response function. On each individual hemisphere we defined PPA, RSC and OPA as the regions responding stronger to places/scenes than to faces blocks in the posterior parahippocampal cortex, in the retrosplenial/parieto-occipital sulcus, and in the transverse occipital sulcus, respectively. The RSC was defined so as to include the posterior cingulate (Brodmann areas 23–31), the retrosplenial cortex proper (Brodmann areas 29–30), and the nearby ventral parietal-occipital sulcus and anterior calcarine sulcus, according to Epstein (2008). We created these ROIs by selecting all activated voxels in the scenes vs. faces contrast (*p* < 0.05 false discovery rate (FDR)-corrected at the cluster level) at a maximum distance of 16 mm from the activation peak. Additionally, for each scene-selective ROI we selected the most responsive 100 cortical nodes, so that all regions contain the same number of nodes, thus allowing us to perform comparisons among them (for a similar procedure, see Vass and Epstein, 2017). All these ROIs were successfully identified in all participants, except for the RSC that was identified in 31/34 hemispheres.

**Figure 3:**
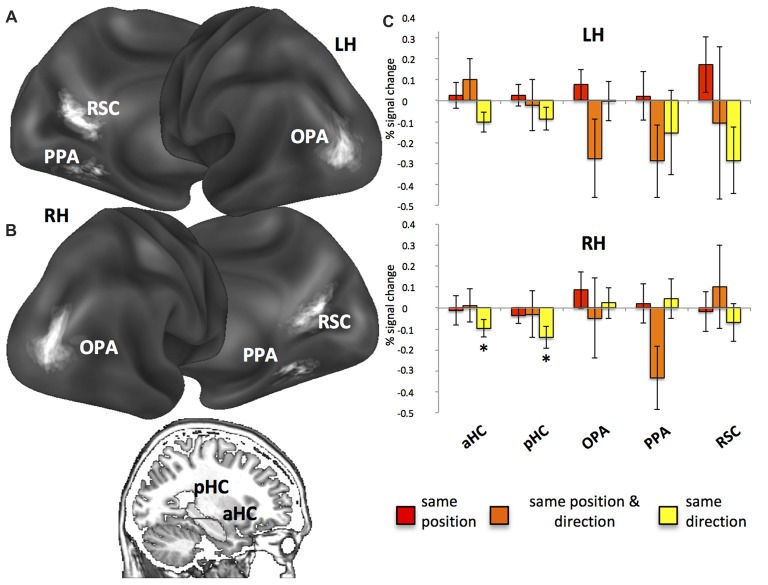
Regions of interest (ROI) and fMR adaptation. **(A)** Anatomical localization of the occipital place area (OPA), parahippocampal place area (PPA) and retrosplenial complex (RSC) on lateral and medial/inferior views of the cortical surface of the left (LH) and right (RH) hemispheres. **(B)** Anatomical localization of the anterior (aHC) and posterior hippocampus (pHC) for one sagittal slice. **(C)** Plots show position- and direction-related adaptation effects, i.e., reduction of estimated BOLD signal in *same position*, *same direction* and *same position & direction* trials as compared to *all-different* trials. *p < 0.05.

A fourth region of interest, the HC, was instead anatomically defined: the automatic segmentation provided by FreeSurfer (Van Leemput et al., 2009) was used to reconstruct the HC of each participant so as to include all CA fields and the subiculum but not the entorhinal cortex. According to Morgan et al. (2011) we further divided each individual HC into an anterior (aHC) and a posterior (pHC) ROI based on an axial division at *z* = −9. The rendering in Figure 3A was created by projecting individual scene-selective ROIs onto a surface-based atlas (Conte69 atlas, Van Essen et al., 2012) using an in-house Matlab toolbox (BrainShow). Figure 3B shows the anatomical localization of aHC and pHC ROIs on a sagittal slice. Table 1 reported MNI coordinates of regional peaks and size of each ROI.

**Table 1:** Regional peaks (MNI coordinates) and size of the regions of interest (ROIs).

Region	Hemisphere	MNI coordinates			Area/Volume
		*x*	*y*	*z*	
aHC	Left	−25	−9	−32	4673 mm^3^
	Right	27	−8	−32	4579 mm^3^
pHC	Left	−29	−37	−9	1666 mm^3^
	Right	33	−36	−9	1597 mm^3^
PPA	Left	−29	−54	−11	114 mm^2^
	Right	29	−47	−10	132 mm^2^
RSC	Left	−20	−62	13	186 mm^2^
	Right	22	−56	13	246 mm^2^
OPA	Left	−32	−91	8	360 mm^2^
	Right	38	−84	10	453 mm^2^

*aHC, anterior hippocampus; pHC, posterior hippocampus; PPA, parahippocampal place area; RSC, retrosplenial complex; OPA, occipital place area*.

For the main experiment analyses, we modeled each trial as a canonical hemodynamic response function time-locked to the trial onset. We included separate regressors for each trial type, thus yielding parameter estimates for the average hemodynamic response evoked by each. Different features of the images presented in each trial were modeled through different GLMs. We modeled target trials with no response (2% on average across subjects), false alarms (1% on average across subjects) and catch trials as separate conditions; these conditions were then excluded from further analyses. All models were fitted to regional time courses from each subject-specific ROI, obtained by averaging preprocessed voxel time series across all voxels within each ROI. Further details about these analyses are reported in the following paragraphs.

### fMR Adaptation Analysis

This analysis was aimed at showing the presence of a neural representation of position and direction in each ROI, based on the fMR adaptation phenomenon, i.e., a reduction of the event-related BOLD signal amplitude to the second trial produced by the repetition of the same position/direction across consecutive trials. This reduction should occur only in brain regions showing selectivity for encoding the repeated information. To this aim, we modeled each target trial with respect to its relationship with the previous target trial in terms of same/different position and direction, thus resulting in the following condition labels: (1) *same position*, for pictures taken from the same wedge of the square as the previous trial, although with a different direction; (2) *same direction*, for pictures taken along the same direction within the square as the previous trial, but from a different place; and (3) *same position and direction*, for pictures taken from the same wedge and in the same direction with respect to the previous trial; and (4) *all different* for pictures taken both from a different position and direction as compared to the previous trial. Note that the pictures shown on successive trials were never exactly identical, even on *same position and direction* trials. Indeed, pictures taken from the same wedge of the square were considered as having the same position although they were taken from two distinct (close) positions. Figure 1B shows an example of trial sequence and the corresponding condition labels.

To examine the neural adaptation effects, beta values associated to each repetition regressor were extracted for each individual ROI, converted to percent signal change, and compared to *all different* condition using one-tailed *t*-tests. We used a FDR procedure (Benjamini and Hochberg, 1995) in order to correct for multiple comparisons: the obtained distributions of *p* values were used to compute a *p* threshold that set the expected rate of falsely rejected null hypotheses to 5%. This procedure was applied for all the subsequent analyses, except when differently specified.

### Multivariate Pattern Analysis

A second way to determine the presence of a neural “signature” of position and direction was based on a MVPA. This is a largely employed classification method in which multi-voxel patterns were classified so as to determine the stimulus category (for a review, see Norman et al., 2006). By training a classifier to discriminate between multi-voxel patterns of estimated BOLD responses elicited by pairs of positions and directions, we aimed at demonstrating the existence of a neural signature of these two spatial information. Specifically, classification accuracies in the analyzed regions were taken as evidence of the presence of position- and direction-related information when significantly higher than chance level.

In the MVPA, we used a GLM on unsmoothed time series, and modeled trials related to each of the six positions and five directions by using separate regressors, in order to estimate the amplitude of the response at each of the 30 trial types across all repetitions. Note that pictures taken along the A direction (catch trials) were excluded from all the analyses. We then used the resulting parameter estimate images to extract multi-voxel pattern of activity for each item in each ROI and classification was performed on these data separately for positions and directions. For each of these two information, we assigned each picture to one of the possible categories, representing the six different positions or the five different directions. The overall classification procedure consisted in splitting the imaging data into two parts: a “training” set used to train a linear classifier (support vector machine (SVM); Duda et al., 2001) using the LIBSVM implementation (Chang and Lin, 2011) to identify patterns of activity related to the stimuli being discriminated, and an independent “test” set used to probe the classification accuracy. We tried to minimize the cross-validation loss during classification by using an automatic Bayesian procedure (as implemented in Statistics and Machine Learning Toolbox^™^ in Matlab R2017b), which chooses a typical set of hyperparameters to optimize. Hyperparameters are internal parameters of the support vector machine that can strongly affect the performance. We used a leave-one-out cross-validation procedure in which data from all scans expect one were used in turn to train the classifier and the remaining scan was used to estimate prediction accuracy. These resulting classification outcomes were then averaged across cross-validation folds and category pairs. We finally used one-sample *t*-tests to compare the between-subject distribution of classification outcomes with chance level (i.e., 0.5). We also compared the classification performance for both position and direction in each ROI through repeated measure ANOVAs. We conducted two separate *spatial information* × *ROI* analyses, one for surface-based ROIs (PPA, RSC and OPA) and one for the anatomical hippocampi (aHC and pHC). The rationale to separate the two ANOVAs is to avoid spurious effects due to the ROI selection procedures, that is functional mapping for surface-based ROIs and anatomical segmentation for the hippocampi.

### Distance-Related Adaptation Analysis

We further looked at the fMR adaptation to explore distance-related effects, i.e., whether neural codes for position and direction reflected real distances between these spatial features. We thus explored whether adaptation effects elicited by consecutive pictures depended on the spatial differences between them. For each of the two spatial information, we used parametric modulators of the BOLD response to model the physical distances between the current and the preceding trial. For this analysis, we modeled all target trials associated with a valid response and preceded by a target trial with a valid response as trials of the same type. This allow us to explore the linear modulation of the response amplitude elicited by both position and direction distances (see below). Target trials associated with missing responses, or following a fixation period, catch trials and false alarms, were modeled separately and not considered here.

We considered two modulatory variables, i.e., position change and direction change, modeling the spatial distance in terms of position and direction, respectively. We considered two estimates of distance for position change, reflecting both the angular and the Euclidean distance between the position (the wedge of the square) from which the current and the preceding trial pictures were taken. Direction change reflects the angular displacement between the allocentric directions of the current and the preceding trial pictures. A third modulatory variable was included to control for the potential confound of visual similarities between the current and the preceding picture (texture change). We introduced this parameter according to Epstein and Morgan (2012) since fMR adaptation could reflect low-level similarities between pictures, irrespective of spatial differences. We used a texture model (Renninger and Malik, 2004) to compute the texture change between each pair of pictures. More specifically, each picture was converted to gray-scale and passed with V1-like filters to create a list of the 100 most prototypical texture features found across the pictures (MATLAB code available at renningerlab.org). For each picture we then generated a histogram of texture frequency. We thus calculated the visual similarity between pairs of pictures by comparing the distribution of the two histograms by using a chi square measure (smaller chi square values correspond to more similar pictures).

To test the hypothesis about the presence of distance-related effects, beta values associated to each modulatory variable were extracted for each individual ROI, converted to percent signal change, and compared to zero using one-tailed *t*-tests.

### Searching for Individual Differences in Position and Direction Coding

Corollary to our main aims, we also checked for the potential link between position- and direction-based representations and individual differences in spatial abilities.

To control for the potential impact of the *a priori* knowledge of the environment on the automatic activation of position- and direction-dependent representations (for a similar procedure, see Sulpizio et al., 2017), we calculated Pearson’s correlations between the behavioral/imaging results and the previous familiarity of the square as assessed, on each participant, by the preliminary questionnaire (see the “Apparatus and Procedure” paragraph).

To test whether position- and direction-related effects depended on the individual ability to build the cognitive map of the square, we calculated the correlation between the behavioral/imaging data with: (1) participants’ scores to the paper-pencil questionnaire assessing the stability of the mental imagery of the square; and (2) the number of runs needed to achieve the supra-threshold accuracy during both position and direction questions of the preliminary training task (see the “Apparatus and Procedure” paragraph). One participant was excluded from this latter analysis due to his deviant data during the position questions, differing by 2.5 standard deviations from the group average. Since any position- and direction-related effects observed during the main task could be due to the ability to memorize positions and directions during the training task, we performed multiple linear regression analyses using the quantity of practice prior to scanning (numbers of runs needed to achieve the criterion in a preliminary training task) as a predictor and the neural effects observed during scanning as the dependent variable. An additional multiple regression analysis was conducted to test whether the observed effects during scanning could be due to the initial accuracy (mean accuracy during the first run) in memorizing positions/directions during the training task rather than by the learning practice.

Finally, to examine the hypothesis that the patterns of activity of scene-selective and hippocampal regions may reflect individual differences in spatial orientation (Sulpizio et al., 2016a), we analyzed the obtained data as a function of the individual navigational abilities as assessed by the SBSOD questionnaire (Hegarty et al., 2002), which is a self-report measure that has been shown to strongly reflect the actual navigation ability thus becoming increasingly used as a reliable instrument to predict real-world wayfinding performance (Janzen et al., 2008; Wegman and Janzen, 2011). For each ROI, participants were divided into two groups (good and poor navigators) by a median split of their SBSOD scores (good group mean 61.22, s.d. 9.28; poor group 47, s.d. 5.76), according to previous reports (Auger et al., 2012; Auger and Maguire, 2013; Wegman et al., 2014; Sulpizio et al., 2016a). We explored the difference between good and poor navigators through a series of mixed analysis of variance (ANOVA), with *group* as a between-subjects variable and *spatial information* (position and direction) as a repeated measure. For these analyses, we used a Bonferroni adjustment in order to create confidence intervals for all the pairwise differences between good and poor navigators.

The analyses on aHC, pHC, OPA and PPA were conducted on 16 participants, eight for each group. One individual was excluded because his score corresponded to the median value to the SBSOD questionnaire. Similarly, the analyses on the right RSC were conducted on 16 participants, eight for each group, because we failed in identifying this area in one participant. Finally, the analyses on the left RSC were conducted on 14 participants, seven for each group. We failed in defining the area in two individuals and one more individual was excluded because his score corresponded to the median value to the SBSOD questionnaire.

For all the above-mentioned analyses, we used both adaptation and decoding results to assess, in each participant, the selectivity for position- and direction-based representations. Specifically, for what concerns the adaptation data, we calculated the difference between repeated and non-repeated (all different) trials on both behavioral and imaging data as the index of the amount of behavioral/neural attenuation.

## Results

### Priming for Repeated Positions: Insight From Behavior

On each stimulus, we asked participants to press a button, except for pictures taken from a specific facing direction (catch trials). This task prompted participants to pay attention to each picture and required them to go beyond the simple analysis of the perceptual features of the scene. Participants performed this task rapidly (median RTs of correct responses: 911 ms; S.D: 154 ms) and quite accurately (Hit: 98%; S.D: 0.3%; FA: 25%, S.D: 12%; and MISS: 0.2%; S.D: 0.3%). Crucially, on each picture, the observer’s position (or facing direction or both) within the familiar place could be same as compared to the preceding trial. In *same position* and in the *same direction* trials, the position and the direction were respectively the same as compared to the previous trial, while the other spatial feature differed. In the *same position and direction* trials, both the position and the direction were the same as compared to the previous trial, while in *all different* trials neither the position nor the direction was the same as the previous trial. We used a series of one-tailed *t*-tests to compare repeated trials to a (common) non-repeated condition. Although participants were not aware of trials repetition, we reported a significant reduction of reaction times (*T*_16_ = −8.13; *p* < 0.0001) for the *same position* (median: 868 ms, S.D: 151 ms) as compared to *all different* trials (median: 903 ms, S.D: 150 ms), index of an implicit representation of position-related spatial information. No significant differences were found between *same direction* (median: 896 ms, S.D: 154 ms) and *all different* trials (*p* > 0.05) and between *same position and direction* (median: 918 ms, S.D: 187 ms) and *all different* trials (*p* > 0.05). Unexpectedly, we did not find any priming effect in the *same position and direction* trials although a priming effect was observed in the *same position* trials. We could speculate that position- and direction-dependent representations are not independent so that the behavioral priming does not necessarily reflect an “additive” effect. Alternatively, such a pattern of results could be explained if considering that pictures in the *same position and direction* trials were never identical since they were labeled as having the same position although they were taken from two distinct (close) positions (1–2) within each wedge of the square (see Figure 1A). These positions were arranged along each direction so that, in this condition, participants could experience (across consecutive trials) to move forward (from position 1 to position 2) or backward (from position 2 to position 1) along a specific direction. This might induce an illusion of self-motion through the square that might interfere with the automatic encoding of position- and direction-related information.

To check for the potential role played by individual differences on this behavioral advantage, we correlated the participants’ performance during the familiarization/training sessions with the priming amount (calculated as the difference between the response time in repeated vs. all different trials) and analyzed this quantity as a function of the self-reported navigational ability. We found no significant correlations between behavior and both the *a priori* familiarization level (as assessed by the preliminary questionnaire) and the imagery-based paper-pencil test (all |r| < 0.36; *p* > 0.15). Further, no significant correlation was found between the behavioral priming and the quantity of practice (number of runs) required to reach the learning criterion in the preliminary training task (*r* = −0.12; *p* = 0.64). These data indicated that the observed behavioral advantage did not reflect the participants’ global knowledge of the environment. When exploring the potential link between the behavioral priming and the self-reported navigational ability, we found no significant results. Good and poor navigators did not show any difference in the amount of priming effects (no significant interaction, *p* > 0.05).

### Neural Codes for Position and Direction: Insight From fMR Adaptation

fMR adaptation, i.e., the neural activity decrease as a function of stimulus repetition, has been extensively used to probe sensitivity to specific visual item and to understand the nature of the underlying representations (Grill-Spector et al., 2006). For fMR adaptation, and for all the subsequent analyses, we report results from the functionally-defined scene-selective regions, RSC, PPA, OPA and from the anatomically-defined anterior (aHC) and posterior (pHC) hippocampi. We focused on these regions since previous neuroimaging studies have implicated them in navigation (Ghaem et al., 1997; Maguire et al., 1998; Rosenbaum et al., 2004; Spiers and Maguire, 2006; Epstein, 2008; Baumann and Mattingley, 2010; Sherrill et al., 2013; Boccia et al., 2017a), spatial memory (Wolbers and Büchel, 2005; Epstein et al., 2007; Brown et al., 2010; Sulpizio et al., 2013, 2016a), spatial orientation (Vass and Epstein, 2013; Marchette et al., 2014; Sulpizio et al., 2014) and spatial imagery (Boccia et al., 2015, 2017b; Vass and Epstein, 2017).

First, we used fMR adaptation to investigate position- and direction-related representations within our ROIs by comparing repeated trials (*same position*, *same direction, same position and direction*) to a common non-repeated condition (*all different*). Results from fMR adaptation are shown in Figure 3 (for a more detailed description about the data distribution, see also Supplementary Figure S2). We observed significant neural adaptations in the right aHC (*t*_16_ = −2.36; *p* < 0.05; Figure 3B) and pHC (*t*_16_ = −2.68; *p* < 0.05) in the *same direction* trials. Significant but FDR-uncorrected results were also observed in the left aHC (left: *t*_16_ = −2.18; *p* = 0.02 uncorrected, corresponding to *p* = 0.07 FDR-corrected) in the *same direction* trials and in the right PPA (*t*_16_ = −2.26; *p* = 0.02 uncorrected, corresponding to *p* = 0.06 FDR-corrected; Figure 3B) in the *same position and direction* trials.

For what concern the individual differences, when controlling for the relationship between adaptation effects and the degree of individual knowledge of the environment, we found some significant (but FDR-uncorrected) correlations: position-related neural effects (calculated as the difference between the neural signal in repeated vs. no repeated trials) was positively correlated with the number of runs required to reach the criterion in the position questions of the training task. This effect was found only in the bilateral PPA (Supplementary Figure S3; left: *r* = 0.54; *p* = 0.031 uncorrected, corresponding to *p* = 0.18 FDR-corrected; right: *r* = 0.61; *p* = 0.013 uncorrected, corresponding to *p* = 0.08 FDR-corrected), thus indicating that the individual ability to rapidly encode the covered positions marginally impacts the neural adaptation in this region.

To further explore this relationship, and to better understand whether the ability to memorize positions/directions in the preliminary task significantly predicts the position- and direction-related neural effects observed in the main task, we performed multiple linear regression analyses using the quantity of practice (number of runs needed to achieve the criterion) as a predictor and the neural effect observed during scanning as the dependent variable. We observed that the quantity of practice in the position task significantly predicted the position-related neural effects in the bilateral PPA (left: Beta = 0.54; *T* = 2.39; *p* = 0.031; right: Beta = 0.61; *T* = 2.86; *p* = 0.013). When exploring whether the initial accuracy in memorizing positions/directions affected the neural effects observed in the main task, we failed in finding significant results (all *p* > 0.1), thus indicating that the learning practice, rather than the initial accuracy, interacts with observed neural effects.

No significant correlations were found between neural adaptation and both the *a priori* knowledge and the mental imagery of the environment (all |r| < 0.45; *p* > 0.07). When examining the relationship between the fMR adaption effects and the self-reported navigational ability, we found no significant results in any ROI. Additionally, good and poor navigators did not differ in the amount of fMR adaption effects (no significant interaction, *p* > 0.05).

**Figure 4:**
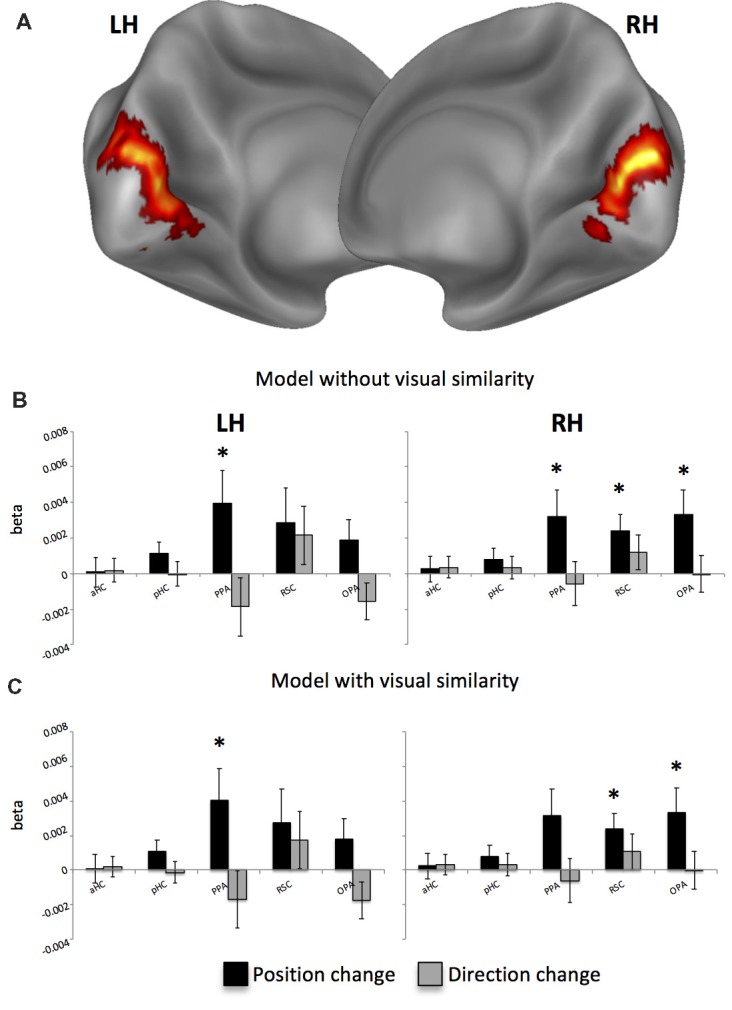
Distance-related results. **(A)** Whole-brain analysis reveals that the visual similarity between consecutive pictures explains activity only in the bilateral early visual cortex (EVC). **(B)** Distance-related adaptation effects without removing variance explained by the visual similarity across pictures. **(C)** Distance-related adaptation effects after controlling for visual similarity across pictures. *p < 0.05.

Second, we asked whether position- and direction-related representations are topographically organized, with neural activity reflecting physical distances between consecutive positions and directions. To do this, we examined distance-related effects on adaptation effects: adaptation between pairs of pictures was taken as an index of the spatial differences between them. A general picture of these effects is shown in Figure 4. When considering the position change as reflecting the angular distance between consecutive pictures, we found a significant positive effect of spatial distances only for position, i.e., an increase of activity as a function of the spatial distances between positions in consecutive pictures, in the bilateral PPA (left: *t*_16_ = 2.21; *p* < 0.05; right: *t*_16_ = 2.16; *p* < 0.05) and in the RSC (*t*_16_ = 2.59; *p* < 0.01) and OPA *t*_16_ = 2.36; *p* < 0.05) of the right hemisphere (Figure 4B). However, to account for the possibility that distance-related neural effects can be due to low-level visual similarity rather than by spatial distance across consecutive pictures, we added a further modulatory variable (texture change). We found that this variable had a significant impact on the early visual cortex (EVC; *p* < 0.001; cluster-level FDR-corrected; Figure 4A), which was the only area reflecting low-level visual change between consecutive pictures. After removing variance due to visual similarity, we confirmed significant linear effects for position-related distances in the left PPA (*t*_16_ = 2.21; *p* < 0.05), in the OPA (*t*_16_ = 2.33; *p* < 0.05) and RSC (*t*_16_ = 2.60; *p* < 0.01) of the right hemisphere (Figure 4C) and marginally in the right PPA (*t*_16_ = 2.27; *p* = 0.02 uncorrected, corresponding to *p* = 0.054 FDR-corrected). These results suggest that visual similarity between consecutive pictures only marginally impacts on distance-related adaptation effects in scene-selective regions.

When examining whether distance-related adaptation effects also reflected real-world Euclidean distances between locations, we found a significant effect in the left PPA (*t*_16_ = 2.49; *p* < 0.05) and in the right RSC (*t*_16_ = 2.29; *p* < 0.05), thus indicating that these regions are sensitive to both angular and metric distances between consecutive positions.

### Decoding the Spatial Information: Insight From Multivariate Pattern Analyses

As a second way to examine the neural codes for position and direction we used multivariate classification analysis. Since visual similarity did not affect the pattern of adaptation results in our ROIs, we did not control for this aspect in the subsequent classification analyses. We also observed that the average visual dissimilarity for between-category image pairs was comparable to the average visual dissimilarity for within-category image pairs (position: between 0.37, within 0.36; *t*_2554_ = −0.86; *p* = 0.19; direction: between 0.37, within 0.37, *t*_2554_ = 1.33; *p* = 0.91) so that no texture-related effect on multi-voxel pattern was expected. We examined the accuracy of a linear classifier in decoding information about position and direction from multi-voxel patterns of neural activity. We obtained independent estimates of neural activity elicited by each of the 30 possible combinations of position and direction, and in separate analyses we grouped the resulting conditions by position and direction. A linear classifier was trained to distinguish between each possible pair of categories (chance level = 0.5) using a cross-validation procedure (leave-one-session-out). More specifically, we tried to decode the specific position (or direction) in each trial from one run from the activity patterns evoked from trials of N-1 runs. For each feature we obtained decoding rates by averaging the decoding performance across all position/direction pairs.

**Figure 5:**
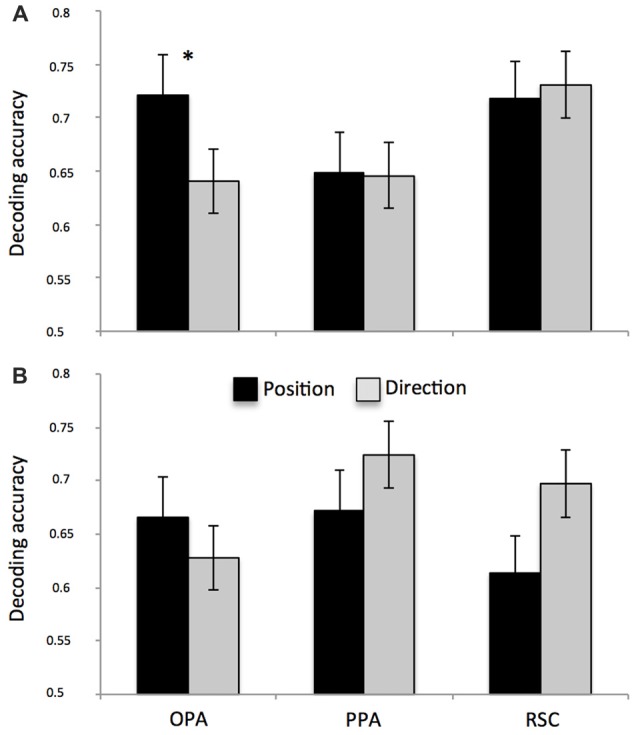
Multivariate classification results. Plots show the mean classification accuracy in the predefined ROIs (all above chance, *p* < 0.01) for both left **(A)** and right **(B)** hemispheres. Classification performance was higher for position than for direction only in the left OPA. *p < 0.01.

We found significantly above-chance decoding accuracy for both position and direction in all ROIs (see Table 2). After directly comparing performance for the two features in each surface-based ROI (Figures 5A,B), we found no differences, except for the left OPA (Figure 5A; *spatial information* by *ROI* interaction, *F*_(2,28)_ = 3.54, *p* = 0.04; $\eta_{\text{p}}^{2}$ = 0.20) in which classification performance was higher for position than for direction (*p* = 0.04). See also Supplementary Figure S4 for a more detailed description about the exact distribution of these data. No significant differences were found in the anatomically-based ROIs, i.e., aHC and pHC.

**Table 2:** Decoding accuracy for both position and direction in the ROIs.

		Position			Direction		
Region	Hemisphere	Mean	SD	*P* value	Mean	SD	*P* value
aHC	Left	0.69	0.14	<0.001	0.66	0.13	<0.001
	Right	0.64	0.14	<0.001	0.64	0.14	<0.001
pHC	Left	0.66	0.17	<0.01	0.71	0.13	<0.001
	Right	0.68	0.17	<0.001	0.69	0.14	<0.001
PPA	Left	0.72	0.15	<0.001	0.64	0.13	<0.001
	Right	0.67	0.16	<0.001	0.63	0.12	<0.001
RSC	Left	0.65	0.16	<0.01	0.65	0.13	<0.001
	Right	0.67	0.15	<0.001	0.72	0.12	<0.001
OPA	Left	0.72	0.14	<0.001	0.73	0.12	<0.001
	Right	0.61	0.13	<0.01	0.70	0.14	<0.001

*aHC, anterior hippocampus; pHC, posterior hippocampus; PPA, parahippocampal place area; RSC, retrosplenial complex; OPA, occipital place area*.

**Figure 6:**
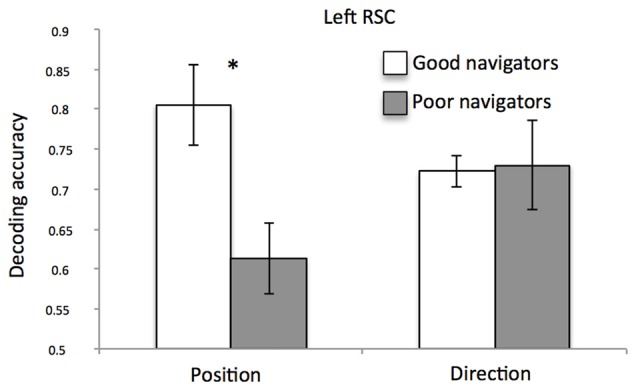
Left RSC decoding accuracy as a function of navigational abilities. Plot shows the mean classification accuracy of the left RSC as a function of group (poor vs. good navigators). *p < 0.01.

When checking for the impact of the individual differences on decoding performances for both position and direction, we found no significant correlations with both the *a priori* knowledge of the environment and the ability to create a stable map of the environment from imagery. However, we found an interesting relationship between classification accuracy and self-reported spatial ability. Figure 6 shows the significant *spatial information* by *group* interaction (*F*_(1,12)_ = 5.76; *p* = 0.03; $\eta_{\text{p}}^{2}$ = 0.32) indicating that, in the left RSC, position-related decoding accuracy was higher for good than for poor navigators (*p* = 0.008; for the exact distribution of these data, see also Supplementary Figure S5).

## Discussion

In the current study, by combining fMR adaptation and multivariate analyses, we set out to ascertain whether the human brain represents spatial information which are relevant for navigation, such as place information about our location in a real, open-field environment and directional information about which orientation we are facing to within it. We sought to clarify whether such a “vista” space is represented in the HC and in scene-selective regions (PPA, RSC and OPA) with the same map-like spatial organization previously observed in a smaller room-sized “vista” environment (Sulpizio et al., 2014), thus getting more light on the impact of the spatial scale on position- and direction-dependent representations.

Finally, we considered how individual differences in navigational abilities and in the ability to build a stable memory of the environment may interact with the function of the navigational system of encoding this two navigationally-relevant information.

Our first main finding is that the right HC contains information about facing direction. This was demonstrated by the finding of neural adaptation for repeated directions: the hippocampal fMRI response to the current trial was reduced by repetition of directional information from the previous trial. The fact that this region did not show neural adaptation for *same position and direction* trials may suggest that the right HC represents directions only when position changes, thus indicating that this area does not simply represents direction. Alternatively, it could be that the *same position and direction* trials were not informative since participants could experience (across consecutive trials) to move forward (from position 1 to position 2) or backward (from position 2 to position 1) along a specific direction, thus interfering with the automatic encoding of position- and direction-based information (see also “Priming for Repeated Positions: Insight From Behavior” paragraph in the “Results” section). Although the hippocampal involvement in directional coding seems to be unusual, this result is in accordance with previous electrophysiological and imaging evidence. For example, a previous report exploring the head-direction system in rats (Golob and Taube, 1999) revealed that lesions to the HC prevent the maintenance of an accurate representation of facing direction. More recently, it has been demonstrated that proximity and orientation toward the goal during a real-world navigation task modulate the hippocampal activity: posterior hippocampal activity increased when participants were close to and facing the goal (Howard et al., 2014). The absence of a position-dependent representation in the HC is not entirely surprising. No position-related fMR adaptation effects were observed in the HC within both virtual room-sized (Sulpizio et al., 2014) and large-scale real environments (Vass and Epstein, 2013). However, the absence of such adaptation effects does not exclude the hippocampal involvement in encoding spatial information about one’s own location. According with previous studies (Hassabis et al., 2009; Sulpizio et al., 2014), we observed that HC accuracy in decoding positions from multi-voxel patterns was significantly above chance, thus indicating that the hippocampal activity contains sufficient information to discriminate different positions. Inconsistent results between fMR adaptation and multi-voxel pattern analysis have been reported before (Drucker and Aguirre, 2009; Epstein and Morgan, 2012). For instance, as suggested by Drucker and Aguirre (2009), these two techniques interrogate representations at different spatial scales: adaptation effects should be more sensitive to the tuning of single (or small populations of) neurons, while multi-voxel effects should reflect clustering distributed at a coarser anatomical scale.

Although we failed in finding adaptation effects in scene-selective regions, clear evidence of a neural “signature” associated with specific spatial locations or directions in PPA, RSC and OPA comes from the multivariate classification analysis. Beyond the HC, we found that the multi-voxel patterns of scene-selective regions contained information about the position and the direction assumed on pictures taken from specific views of the familiar circular real-world square. We previously observed that PPA and RSC contained place-, view- and heading information concerning the scene currently being viewed within a smaller room-sized vista environment, which permitted successful decoding by the classifier (Sulpizio et al., 2014). Our finding that PPA, RSC and OPA represent position and direction confirms previous neuroimaging studies. These regions have been involved previously in spatial navigation and spatial memory (for a recent review, see Epstein et al., 2017). More generally, a recent meta-analysis reported in Epstein et al. (2017) revealed that the common activation across 64 studies of human navigation well corresponded to our ROIs. More specifically, previous studies on multi-voxel pattern analysis revealed that PPA, RSC and OPA contain information about scene category (Kravitz et al., 2011; Epstein and Morgan, 2012) and specific landmarks (Morgan et al., 2011; Epstein and Morgan, 2012) within a large-scale real space and allow classification of interiors from exteriors of buildings (and vice versa) within that environment (Vass and Epstein, 2013; Marchette et al., 2014). Our results extend these previous findings, by showing that all the above-mentioned scene-selective regions contain sufficient information that allow to discriminate different location/direction within a real “vista” space, thus supporting the idea that they are recruited whenever people are exposed to pictures of scenes, independently of both environmental features and task demands.

Analyses of multi-voxel patterns also revealed that OPA is a key region in discriminating different positions within the square: it was able to distinguish (better than chance) different facing directions, but we observed higher decoding performance after applying MVPA to predict distinct locations. Previous research suggests that OPA is causally involved in scene processing (Dilks et al., 2013) and more specifically in the spatial processing of local scene elements such as environmental boundaries (Julian et al., 2016), and that it automatically encodes the structure of the navigational space, by detecting environmental features that afford relevant behaviors such as navigation (Bonner and Epstein, 2017; Patai and Spiers, 2017). The direct role of this area in the human visually-guided navigation has been further supported by previous evidence showing its involvement in supporting obstacle avoidance in the immediately visible scene (Kamps et al., 2016) and in encoding two essential kinds of information: sense (left-right) information and egocentric distance (proximal-distal) information (Dilks et al., 2011; Persichetti and Dilks, 2016). The current work purports to show that OPA represents directions and (especially) positions of vista spaces invariant to these particular scene features. It is possible that different scenes depicting similar positions or directions depict similar boundaries or affordances, but a more careful analysis of the scene content would be required to differentiate these alternatives.

Beyond distinguishing between positions and directions, another key characteristic of scene-selective regions is that they support a sort of “cognitive map” of the environment, i.e., the neural representations reflect the spatial structure of the environment they represent. By examining the fMR adaptation on each trial as a function of the real distances between consecutive positions, we observed that the activity in the bilateral PPA and in the right RSC and OPA scales with these distances: i.e., greater fMRI responses for larger distances. Interestingly, these distance-related effects were observed although participants were not given any explicit navigational task or distance estimation demands, suggesting that these distance-related representations are automatically activated. Importantly, this result cannot be explained by differences of visual features between consecutive pictures. Once explicitly modeled low-level visual similarity between consecutive views, we observed significant effect on the early visual areas only. Compatibly, after removing effects of visual similarity, we obtained the same pattern of adaptation results on scene-selective regions, thus indicating that these regions, in line with previous evidence (Epstein and Morgan, 2012; Sulpizio et al., 2014), do not represent low-level visual properties of the scene.

Further support to the PPA and RSC involvement in encoding distances between locations comes from the observation that the right RSC and the left PPA are sensitive not only to the angular but also to the Euclidean distances between consecutive positions. A similar distance–related effect was recently observed in both PPA and RSC during the exposure to pictures taken from a familiar virtual room (Sulpizio et al., 2014). Both regions exhibited adaptation effects, proportional to the physical distances between consecutive places and views. On the other side, no evidence of a relationship between the activity of RSC and PPA and real-world distances between locations were found in previous studies examining the neural codes of real positions within large-scale environments. We speculate that the critical aspect to be considered when trying to justify this discrepancy is the set of properties of the immediate visible surrounding. It is possible that a metric, map-like representation precisely preserving distance relationships between spatial locations is easier to build up in “vista” spaces, where spatial locations to be encoded are often simultaneously in view during navigation (Wolbers and Wiener, 2014).

Another important aspect we considered is the potential impact of the individual differences on spatial representations. We found a relationship between the amount of practice needed to memorize the covered positions and the fMRI attenuation in the bilateral PPA: the longer the training the participants needed to memorize all positions, the higher the signal (i.e., the lower the neural adaptation) in this region. This result accounts for a link between position-dependent representation in PPA and the individual ability to memorize the covered positions within the environment. Consistently, Epstein et al. (2005), by examining how scene representations vary across individuals as a function of individual differences, previously observed that adaptation effects in PPA was larger for people with higher navigational competence.

The relationship between the individual ability to achieve a long-term memory for locations experienced prior to scanning and the observed position-related neural effects speaks in favor of a significant impact of learning rapidity in the position-dependent representation in PPA. One could argue that the initial predisposition to memorize different positions, which should reflect the individual ability to retrieve locations experienced during the *in loco* navigation session, may account for the observed neural effects. However, we found that the initial accuracy in memorizing locations during the first run of the training task did not predict the position-related neural effects, thus indicating that the amount of practice needed to reach a stable memory of locations, rather than the individual (*a priori*) promptness to memorize different positions, affects the position-related representation in the PPA. The sensitivity to environmental learning observed in the PPA supports previous evidence that has demonstrated the PPA/PHC involvement in rapid learning of specific associations between (initially unfamiliar) scenes (Turk-Browne et al., 2012).

As a further attempt to get more light on the individual differences, we examined how position- and direction-dependent representations may explain individual differences as a function of self-reported navigational abilities. We found an interesting relationship between multi-voxel classification accuracy and self-reported spatial ability in the left RSC: classification accuracy for different positions was higher in good than in poor navigators. This result is in line with a series of previous imaging studies showing the crucial role of RSC in accounting for individual differences in spatial abilities (Auger et al., 2012; Auger and Maguire, 2013; Sulpizio et al., 2016a). For example, poor navigators were impaired at identifying the most permanent items in the environment, and exhibited reduced responses in RSC, as compared to good navigators (Auger et al., 2012). By looking at the multi-voxel activity patterns in RSC, it was observed a better decoding of the number of permanent landmarks in good rather than in poor navigators (Auger and Maguire, 2013); similarly, the resting-state functional connectivity between the posterior HC and RSC was significantly higher in good than in poor navigators (Sulpizio et al., 2016a). An unexpected finding was that the relationship between the RSC activity and individual differences was observed only when spatial abilities were assessed using self-reports rather than more objective experimental measures, such as the above-mentioned preliminary training task. One possible explanation is that, while the SBSOD has been often used as a reliable proxy for real-world navigation performance (Janzen et al., 2008; Wegman and Janzen, 2011), the training task, by focusing on giving participants a long-term knowledge of the environment, tested spatial memory rather than actual navigation abilities. To go beyond this limitation, future studies should benefit in using, besides the subjective self-reported ones, more implicit experimental tasks on actual navigation. Another potential limitation of this study should be considered. The sample size was relatively small, which may limit the statistical power especially when detecting individual differences. Thus, regression and correlation analysis results should be interpreted with caution.

For what concern the hemispheric laterality of the observed effects, we mainly reported right-lateralized results, with the right HC showing neural suppression for repeated directions, and the right RSC and OPA exhibiting distance related adaption effects for consecutive positions. These results confirm the well-established right-hemispheric dominance for spatial tasks (for reviews, see Burgess et al., 2002; Boccia et al., 2014). Additionally, we also found some left-lateralized effects in OPA, which prefers to decode locations than directions, in RSC, whose multivariate pattern in decoding different locations predicts individual differences in spatial ability, and in PPA, in which activity was modulated by both angular and metric distances between consecutive positions. However, particularly for OPA and PPA, no study to our knowledge previously reported hemispheric differences so that the question of hemispheric laterality is still a matter of dispute, and future studies should help to clarify this issue.

In summary, the present findings demonstrated that the human navigational network, including the HC and scene-selective regions, encodes spatial information about location and direction within a real “vista” environment, even in the absence of a navigational task. Furthermore, our results provide new insights into how the navigational network represents a real large-scale “vista” space. In particular we found that scene-selective regions (but not the HC) support a map-like representation of the environment, since they exhibited adaptation effects sensitive to real-world distances between consecutive positions. These results indicate that the neural code for one’s own position and direction within a large-scale circular square is organized at a coarser spatial scale as compared to the metric representation observed in the small-scale, room-size environment (Sulpizio et al., 2014), thus accounting for a feeble impact of the scale of space on these spatial codes within the “vista” space. However, the spatial scale is not enough to differentiate between these two space classes that define the human navigational experience, i.e., “vista” and “environmental” spaces. A recent work, indeed, suggested that spatial memories for locations in “vista” and “environmental” spaces are qualitatively different in terms of spatio-temporal learning experience, and reference frame orientation employed during navigation: contrary to “vista” space, retrieving memory from “environmental” space requires to access to both order and distance in which objects are learned (Meilinger et al., 2016). Thus, further studies should better explore not only the role of the environmental size but also the impact of other variables, such as the amount of information to maintain, distance and order effects, or the alignment of the reference frame on such a map-like representation by directly manipulating the scale of space in both “vista” and “environmental” spaces.

## Author Contributions

VS, MB and GG designed the study. VS and MB collected the data. VS and GG analyzed the data and VS wrote a first draft of the manuscript. VS, MB, CG and GG contributed to the final version of the manuscript.

## Conflict of Interest Statement

The authors declare that the research was conducted in the absence of any commercial or financial relationships that could be construed as a potential conflict of interest.

## Supplementary Material

FIGURE S1 Training task. **(A)** Example of a trial of the training task about position. Participants were instructed to decide whether the position they perceived in the square corresponded to the green wedge on the sketch. The label below the photograph (not shown to the participant) identifies the position from which the photograph is taken (first two letters: 1A to 2F) and its facing direction (third letter: A–F). **(B)** Example of a trial of the training task about direction. Participants were instructed to decide whether the direction they faced at corresponded to the green arm on the sketch.

FIGURE S2 Box plots showing a more detailed distribution of position- and direction-related adaptation effects showed in Figure 3C.

FIGURE S3 Correlations between neural signal and the amount of practice. Scatterplots show the correlations between the neural signal (repeated position *minus* no repeated) and the amount of practice (number of runs needed to reach at least 70% of accuracy in the position questions of the training task) in the bilateral PPA.

FIGURE S4 Box plots showing a more detailed distribution of multivariate classification results showed in Figures 5A,B.

FIGURE S5 Box plot showing a more detailed distribution of the left RSC decoding accuracy result as a function of navigational abilities as shown in Figure 6.
