# Supplementary figures and images for: Neural Codes for One’s Own Position and Direction in a Real-World “Vista” Environment
Source: Front Hum Neurosci. 2018 Apr 30;12:167. doi: 10.3389/fnhum.2018.00167 (PMC5936771; doi:10.3389/fnhum.2018.00167)

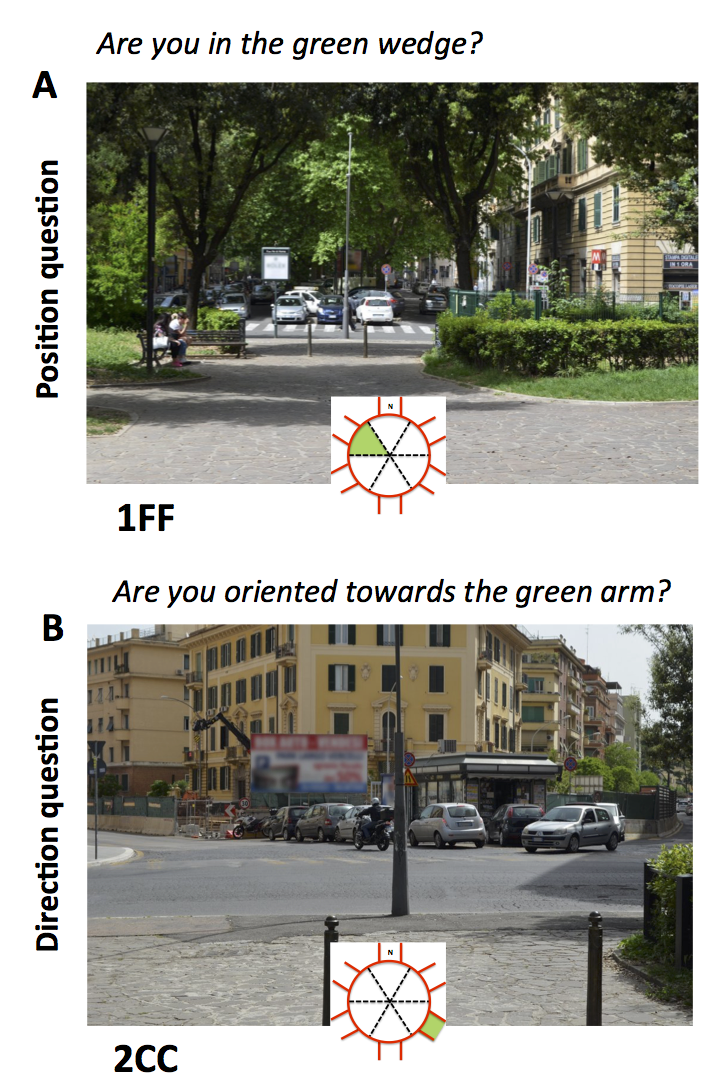

Supplement: FIGURE S1 — Training task. (A) Example of a trial of the training task about position. Participants were instructed to decide whether the position they perceived in the square corresponded to the green wedge on the sketch. The label below the photograph (not shown to the participant) identifies the position from which the photograph is taken (first two letters: 1A to 2F) and its facing direction (third letter: A–F). (B) Example of a trial of the training task about direction. Participants were instructed to decide whether the direction they faced at corresponded to the green arm on the sketch. [file Image_1.TIFF]

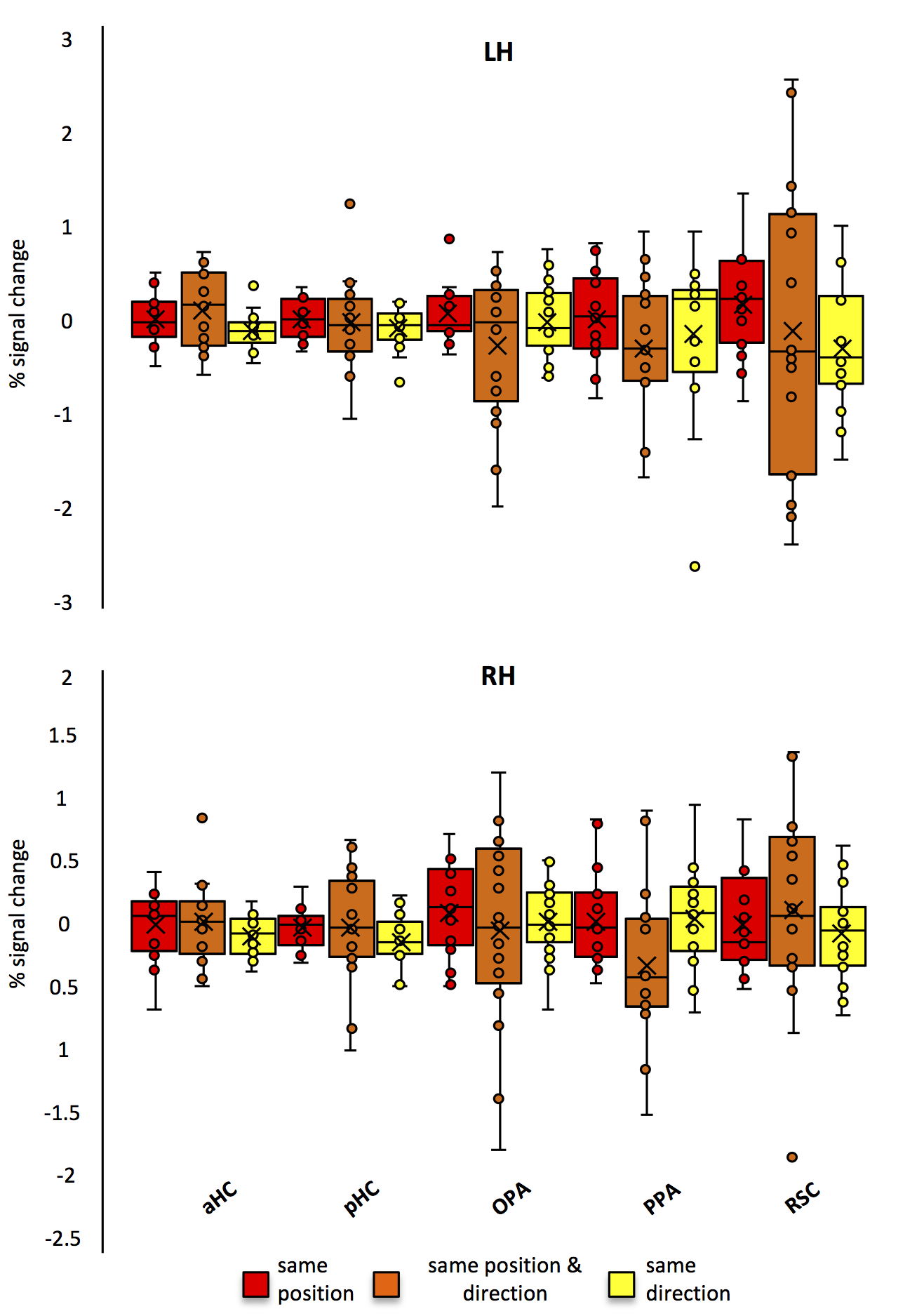

Supplement: FIGURE S2 — Box plots showing a more detailed distribution of position- and direction-related adaptation effects showed in Figure 3C. [file Image_2.TIFF]

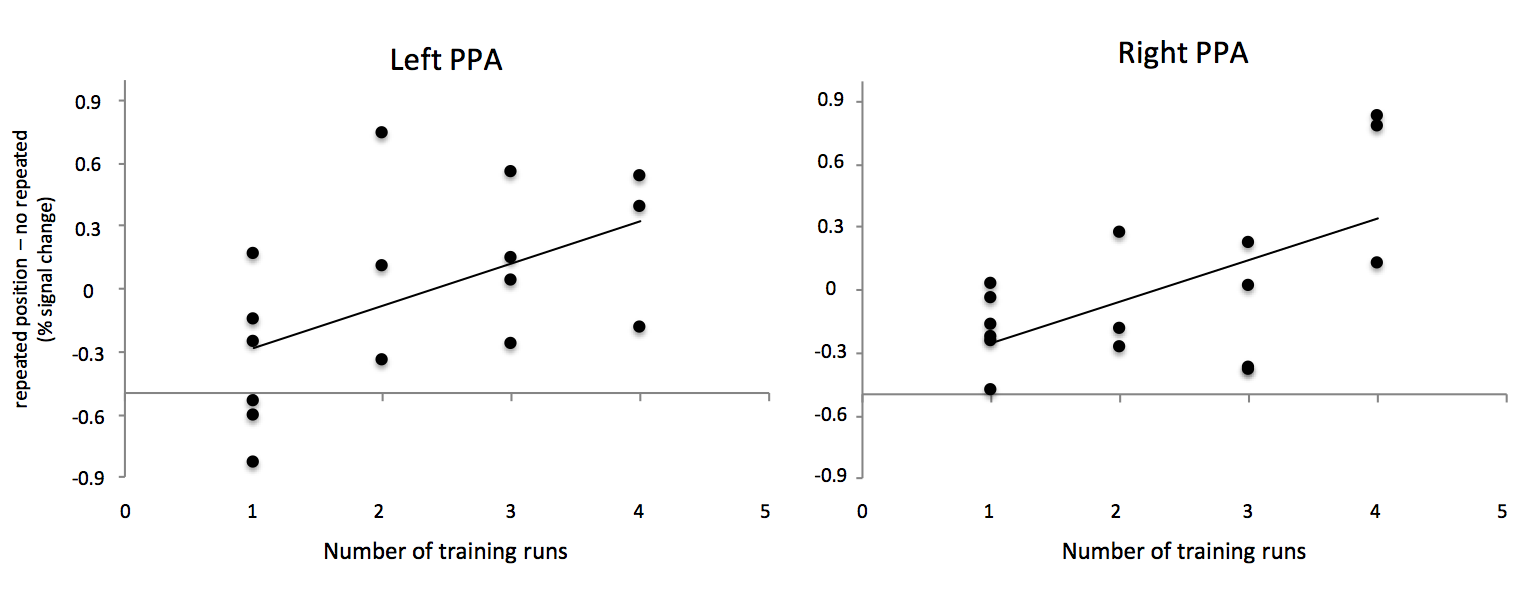

Supplement: FIGURE S3 — Correlations between neural signal and the amount of practice. Scatterplots show the correlations between the neural signal (repeated position minus no repeated) and the amount of practice (number of runs needed to reach at least 70% of accuracy in the position questions of the training task) in the bilateral PPA. [file Image_3.TIFF]

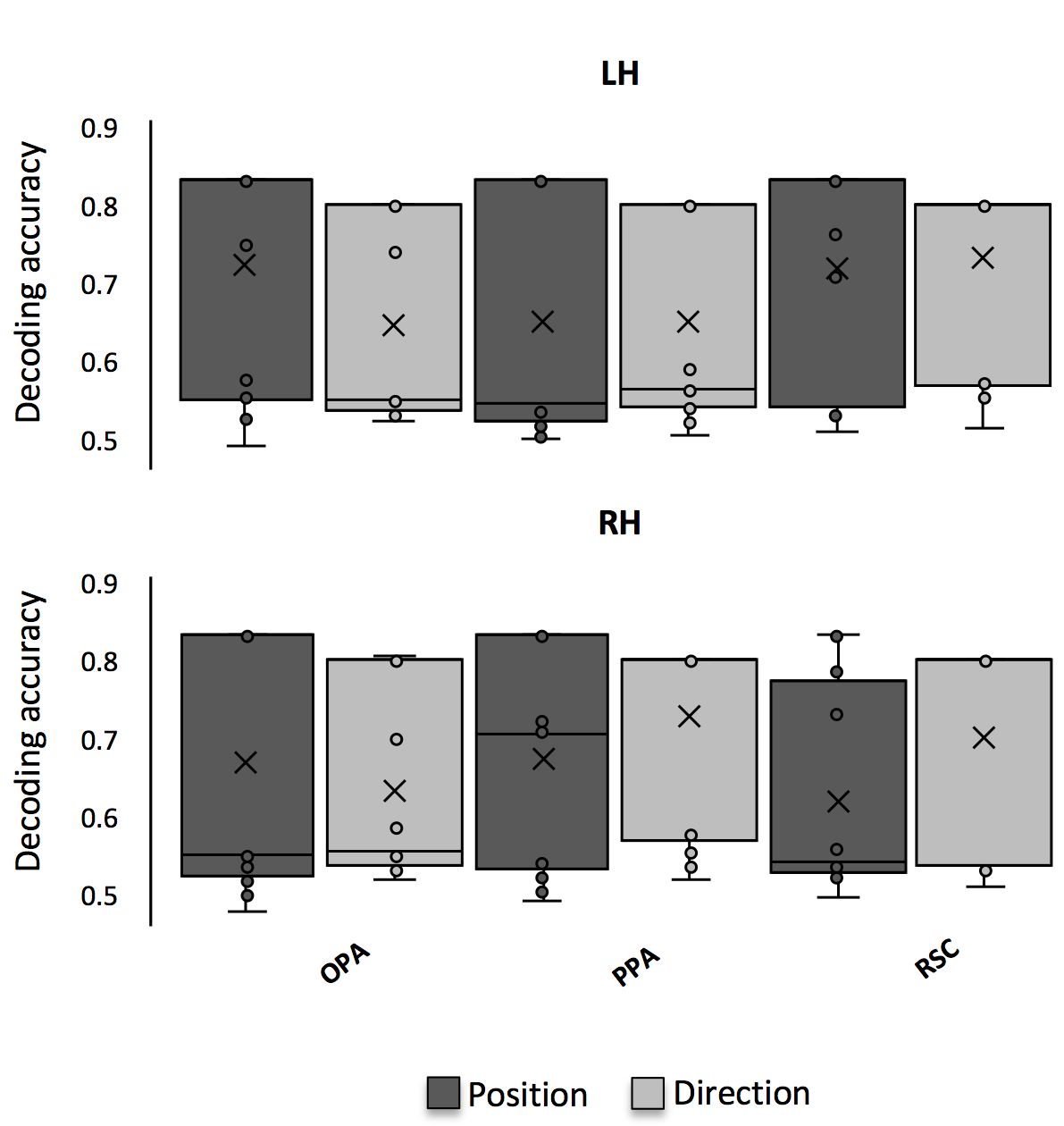

Supplement: FIGURE S4 — Box plots showing a more detailed distribution of multivariate classification results showed in Figures 5A,B. [file Image_4.TIFF]

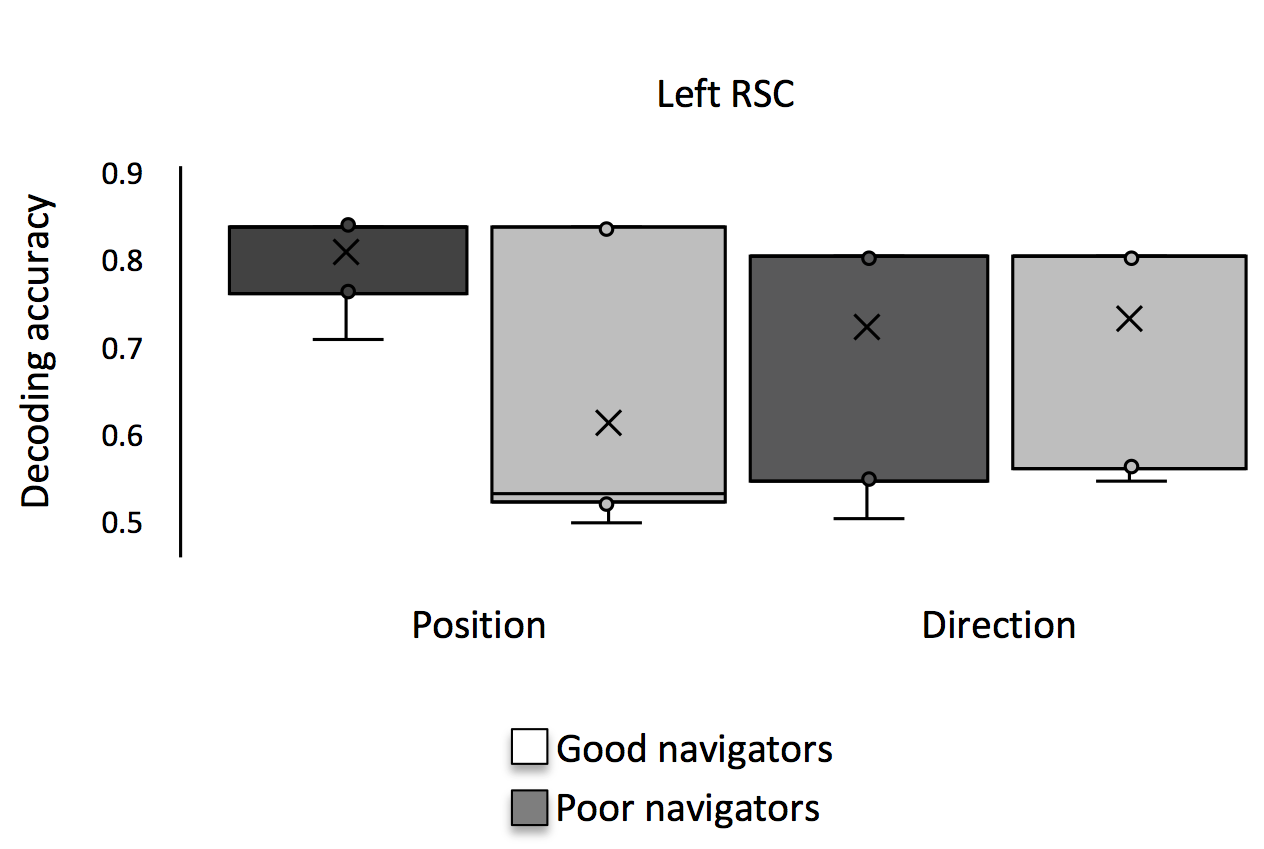

Supplement: FIGURE S5 — Box plot showing a more detailed distribution of the left RSC decoding accuracy result as a function of navigational abilities as shown in Figure 6. [file Image_5.TIFF]
